# Development of a yeast model to study the contribution of vacuolar polyphosphate metabolism to lysine polyphosphorylation

**DOI:** 10.1074/jbc.RA119.011680

**Published:** 2019-12-16

**Authors:** Cristina Azevedo, Yann Desfougères, Yannasittha Jiramongkol, Hamish Partington, Sasanan Trakansuebkul, Jyoti Singh, Nicole Steck, Henning J. Jessen, Adolfo Saiardi

**Affiliations:** ‡ Medical Research Council, Laboratory for Molecular Cell Biology, University College London, London WC1E 6BT, United Kingdom; § Institute of Organic Chemistry, University of Freiburg, 79104 Freiburg, Germany; ¶ CIBSS–Centre for Integrative Biological Signalling Studies, University of Freiburg, 79104 Freiburg, Germany

**Keywords:** phosphorylation, protein phosphorylation, cell signaling, post-translational modification (PTM), molecular cell biology, inorganic polyphosphate, lysine modifications, lysine polyphosphorylation (K-PPn), polyphosphatases, Top1

## Abstract

A recently-discovered protein post-translational modification, lysine polyphosphorylation (K-PPn), consists of the covalent attachment of inorganic polyphosphate (polyP) to lysine residues. The nonenzymatic nature of K-PPn means that the degree of this modification depends on both polyP abundance and the amino acids surrounding the modified lysine. K-PPn was originally discovered in budding yeast (*Saccharomyces cerevisiae*), in which polyP anabolism and catabolism are well-characterized. However, yeast vacuoles accumulate large amounts of polyP, and upon cell lysis, the release of the vacuolar polyP could nonphysiologically cause K-PPn of nuclear and cytosolic targets. Moreover, yeast vacuoles possess two very active endopolyphosphatases, Ppn1 and Ppn2, that could have opposing effects on the extent of K-PPn. Here, we characterized the contribution of vacuolar polyP metabolism to K-PPn of two yeast proteins, Top1 (DNA topoisomerase 1) and Nsr1 (nuclear signal recognition 1). We discovered that whereas Top1-targeting K-PPn is only marginally affected by vacuolar polyP metabolism, Nsr1-targeting K-PPn is highly sensitive to the release of polyP and of endopolyphosphatases from the vacuole. Therefore, to better study K-PPn of cytosolic and nuclear targets, we constructed a yeast strain devoid of vacuolar polyP by targeting the exopolyphosphatase Ppx1 to the vacuole and concomitantly depleting the two endopolyphosphatases (*ppn1*Δ*ppn2*Δ, vt-Ppx1). This strain enabled us to study K-PPn of cytosolic and nuclear targets without the interfering effects of cell lysis on vacuole polyP and of endopolyphosphatases. Furthermore, we also define the fundamental nature of the acidic amino acid residues to the K-PPn target domain.

## Introduction

One of the major challenges of the post-genomic era is to decode the molecular map of all protein post-translational modifications (PTMs)^4^ controlling normal physiology and disease development. These modifications increase the structural and functional complexity of proteins allowing for a rapid, controlled, and often reversible response to internal cues and environmental signals. Hundreds of PTMs have been identified to date, and many keep being discovered (1). PTMs can be broadly classified into two categories: enzymatic, in which a second protein is actively modifying a client peptide, and nonenzymatic, which relies on the chemical features of amino acid side chains and of reactive metabolites. The specificity of enzymatic PTMs, like serine or threonine phosphorylation for example, derives mainly from the enzymatic selectivity of the specific kinases able to recognize target proteins and sequence motifs on their clients. In contrast, nonenzymatic PTMs occur without the involvement of a protein catalyser, through spontaneous chemical reactions between amino acid functional groups and reactive metabolites (2). The spontaneous nature of nonenzymatic PTMs does not mean that these occur randomly: there are rules dictating the selectivity and signal strength of nonenzymatic PTMs. In recent years, significant progress has been made in deciphering the rules governing cysteine *S*-nitrosylation, one of the best-characterized nonenzymatic PTMs. These studies indicate that the probability of a specific cysteine to be *S*-nitrosylated depends on its biochemical and/or structural properties, *i.e.* the p*K_a_*, solvent exposure, its location in α-helices, and the close presence of positively-charged residues (3). Moreover, the degree of *S*-nitrosylation depends primarily on the abundance of the “reactive metabolite,” nitric oxide (NO), and therefore on the location and the regulated activity of its synthesizing enzyme, the NO-synthase (4). The same rules are likely to be applied to all nonenzymatic PTMs, with the selectivity dictated by the chemical and physical surroundings of the targeted amino acids and the signal strength controlled by the concentration of the specific reactive metabolite. One such PTM is the modification of lysine polyphosphorylation (K-PPn) that consists of the covalent attachment of inorganic polyphosphate (polyP), a ubiquitous linear polymer of phosphate residues, to target proteins (5). Polyphosphorylation occurs at lysine residues, creating a phosphoramidate bond (P–N) that, unlike phosphoester bonds, is highly labile under the acidic conditions typically used in mass spectrometry (MS). Lysine is an electron-rich positively-charged amino acid with a nucleophilic side chain, making it one of the most modified amino acids in nature (6). Together, these characteristics make the thorough characterization of K-PPn a challenging endeavor. Two K-PPn target proteins have been characterized in detail so far, the nuclear signal recognition 1 (Nsr1), a protein involved in ribosomal rRNA biogenesis (7), and its interacting partner topoisomerase 1 (Top1), an enzyme that unwinds supercoiled DNA. K-PPn leads to an increase in molecular weight as seen by an upwards mobility shift on NuPAGE SDS gels. The target lysine residues are located within the PASK domain (polyacidic serine and lysine(K)), a region characterized by an abundance of acidic amino acids (glutamic and aspartic acids) and lysine and serine residues (5). The importance of these amino acids for the modification is poorly understood. It has been demonstrated that mutations of all glutamic acid, aspartic acid, or serine residues within the PASK domain do not directly affect Top1 K-PPn (5). However, deletion constructs within the PASK domain of Top1 significantly reduced the mobility shift of this domain, suggesting that the three-dimensional structure of the PASK domain might play an important role in dictating the degree of the modification and/or that there are several lysine targets within the PASK domain.

All organisms from bacteria to mammals possess polyP. This simple polymer was discovered more than a century ago and for a long time was believed to be simply a phosphate storage molecule. However, recently it was revealed that polyP is a multifaceted molecule possessing several functional roles in numerous biological contexts. Among some of the functions ascribed to polyP are as follows: its chaperone-like properties for stress protection (7), its role as a blood coagulation factor (8, 9), and it is a modulator of infectivity of bacterial and protozoan pathogens (10, 11). The polyP biosynthetic pathway is characterized in bacteria, yeast, *Trypanosoma,* and *Dictyostelium*. In mammalian cells, despite many reports showing the existence of polyP, no homologues of PPK1 and PPK2 (polyP kinase 1 and 2, respectively) from prokaryotes or vacuolar transport complex (Vtc) from protists have been found to date. *Dictyostelium* possesses a PPK1 bacterial-like enzyme acquired by horizontal gene transfer (12), whereas trypanosomes possess Vtc4 yeast-like enzymology (13).

The fact that in budding yeast all the enzymology for the synthesis and hydrolysis of polyP is known makes this the preferred model in which to study K-PPn. However, this model has some drawbacks. *Saccharomyces cerevisiae* has very high concentrations of polyP, 80% of which is located in the vacuole (5) and therefore is not physiologically available to K-PPn cytosolic or nuclear proteins. Moreover, yeast possess four polyP-phosphatases able to hydrolyze polyP. Three are endopolyphosphatases, enzymes that hydrolyze polyP internally, namely Ppn1 (14, 15), Ppn2 (16), and Ddp1 (17). A very active exopolyphosphatase is also present, Ppx1, an enzyme that hydrolyzes polyP from the terminal phosphate, to release P_i_ and PP_i_ as the final products (18). Ppn1 and Ppn2 have a strict vacuole localization. Ppx1, Ddp1, and Ppn1 not only target “naked” polyP but are also known to actively de-polyphosphorylate proteins (5).

The nonenzymatic nature of K-PPn predicts that the degree of this modification can be influenced by the abundance of polyP. Therefore, during cell lysis, the abundant polyP of the vacuole is released from the broken organelle and could subsequently nonphysiologically attach to target proteins. This is an issue common to most nonenzymatic PTMs where the reactive metabolite and the target protein can come in contact during cell lysis. K-PPn analysis also encounters the opposite problem, the release of the polyP phosphatases Ppn1 and Ppn2 from the vacuole. Their release in the cell lysate could result in a reduction of the degree of K-PPn. Here, we investigate these hypotheses leading to a better characterization of K-PPn, and we report on the creation of a budding yeast strain suited to study this modification in a more physiological context.

## Results

### Polyphosphorylation mobility shift can be exacerbated during cell lysis

**Figure 1.**
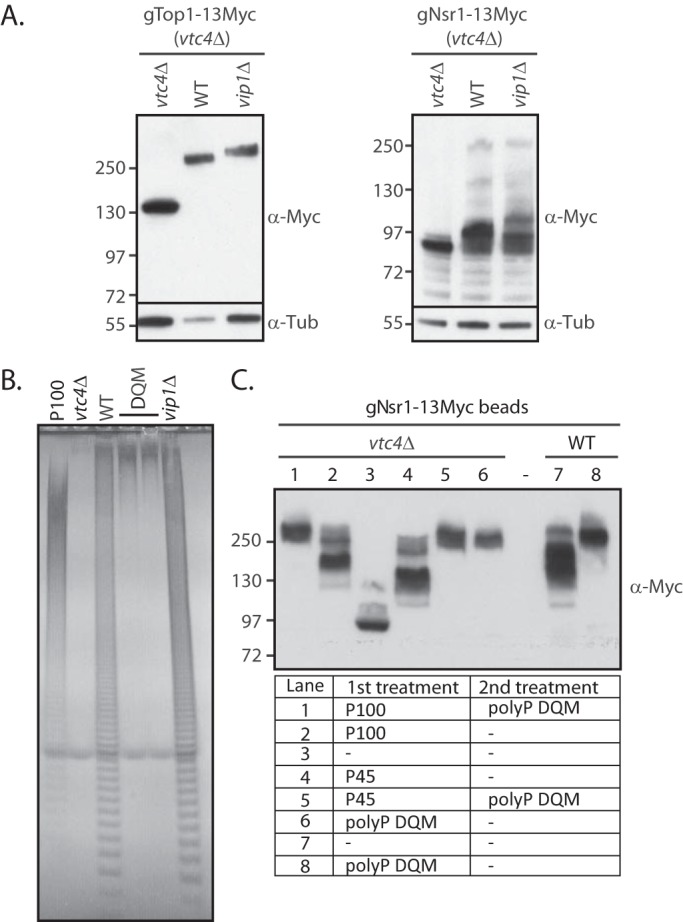
**Polyphosphorylation can be exacerbated during the extraction procedures.** *A,* cultures from DDY1810 yeast containing different levels of polyP (no polyP-*vtc4*Δ, normal polyP-WT, and high polyP-*vip1*Δ) were mixed in a 1:1 ratio (OD_600_ = 0.8) with a culture from gTop1–13Myc- or gNsr1–13Myc-tagged *vtc4*Δ; the proteins were extracted, run on NuPAGE, and blotted with anti-Myc to detect Top1 and Nsr1 and with anti-tubulin (α-*Tub*) (loading control). *B,* total polyP from the *ppn1*Δ*ppn2*Δ*ppx1*Δ*ddp1*Δ quadruple DDY1810 mutant (DQM). PolyP corresponding to 5 μg of total RNA was fractionated on a 30% polyacrylamide gel and stained by negative staining with DAPI. *C,* shift-up experiment of purified gNsr1–13Myc (*vtc4*Δ), kept on Myc-agarose beads, subjected to several treatments, in short, the beads were incubated for 20 min at 30 °C either with 2 mm polyP100 (*P100*), polyP45 (*P45*), polyP extracted from the *ppn1*Δ*ppn2*Δ*ppx1*Δ*ddp1*Δ DDY1810 quadruple mutant (*DQM*) or left untreated, washed in lysis buffer, and further incubated with polyP as described in panel *C*. Protein samples were run on NuPAGE and blotted with anti-Myc. The figures presented are a representation of at least three independent repeats.

The nonenzymatic nature of K-PPn made us question whether upon extraction there could be an exacerbation of the modification, as measured by a mobility shift on NuPAGE. We first tested whether the polyP released from the vacuoles could interact with proteins, altering their mobility on NuPAGE, by mixing two cell cultures in a 1:1 ratio, one devoid of polyP (*vtc4*Δ) and the other possessing polyP. PolyP-deficient *vtc4*Δ cells expressing Myc-tagged Top1 or Nsr1 (gTop1–13Myc and gNsr1–13Myc, respectively) were mixed with a second culture containing either normal (WT), high levels (*vip1*Δ), or no polyP (*vtc4*Δ). Vip1 is a yeast diphosphoinositol pentakisphosphate kinase whose deletion is known to result in elevated polyP levels (19). The proteins were immediately extracted, as described under “Experimental procedures,” without detergent and using glass beads for physical homogenization. The predicted molecular masses of 130 kDa for gTop1–13Myc and 80 kDa for gNsr1–13Myc were observed, as expected, from extracts containing no polyP (*vtc4*Δ + *vtc4*Δ; Fig. 1*A*). However, the upward gel-mobility shift characteristic of K-PPn was observed for both proteins in extracts where polyP was present (*vtc4*Δ + WT; *vtc4*Δ + *vip1*Δ). This result confirms the hypothesis that K-PPn can occur during cell lysis. Next, we assessed whether K-PPn proteins could be further polyphosphorylated post-extraction. To assess this, we used polyP of two different lengths. One was synthetic polyP of an average chain length of 100 phosphate groups, herein called P100. To make polyP with an average chain length of longer than 100, we used a yeast quadruple mutant in which all the polyP phosphatases are deleted (*ppn1*Δ*ppn2*Δ*ppx1*Δ*ddp1*Δ), herein called DDY1810 QM or simply DQM (Fig. 1*B*). Protein extracts from gNsr1–13Myc *vtc4*Δ (Fig. 1*C*, *lane 3*) were first incubated with P100 (Fig. 1*C*, *lane 2*), followed by incubation with extract from the DQM mutant. This experiment revealed that the mobility of gNsr1–13Myc can be further increased after the first K-PPn modification (Fig. 1*C*, *lane 1*). The same was observed when non-K-PPn gNsr1–13Myc treated with synthetic polyP of average chain length 45 (herein called P45) was subsequently mixed with DQM extract (Fig. 1*C*, *lane 4*). Importantly, gNsr1–13Myc from WT yeast, endogenously K-PPn, also shifted up after mixing with DQM extract (Fig. 1*C*, *lanes 7* and *8*). These results indicate that K-PPn proteins can be further polyphosphorylated during cell lysis.

### Polyphosphorylation has an opening effect on target proteins

**Figure 2.**
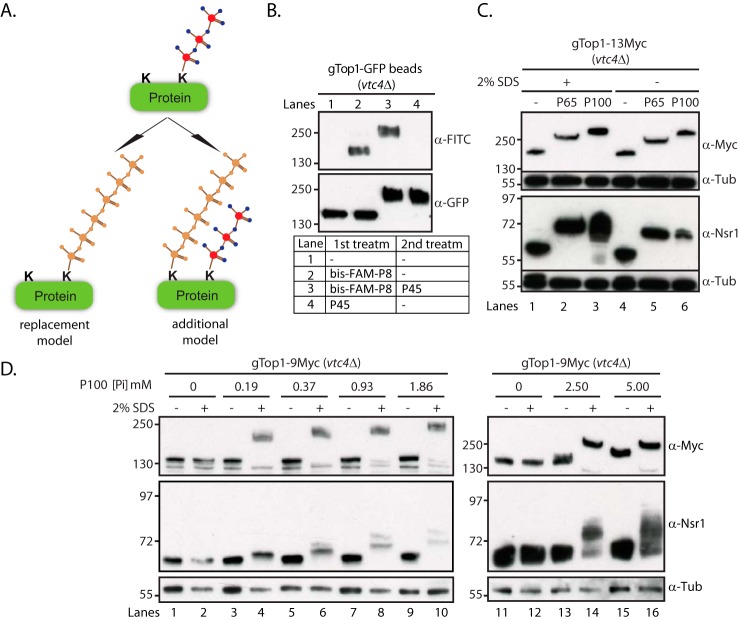
**Polyphosphorylation effect on Top1 and Nsr1 mobility shift.** *A,* schematic representation of the putative models for K-PPn mobility shift enhancement. The replacement model suggests that the polyP in a K-PPn target protein can be substituted on the same lysine residue by the additional polyP. The additional model suggests that polyphosphorylation, exposes buried lysine residues, can then be *de novo* polyphosphorylated once further polyP is available. *B,* testing the replacement model. Shift-up experiment of purified unpolyphosphorylated gTop1–13Myc (*vtc4*Δ) was kept on Myc-agarose beads and subjected to several treatments; in short, the beads were first incubated for 20 min at 30 °C either with 2 mm P45 or 0.5 mm bis-FAM-P8 or left untreated, washed in lysis buffer (see under “Experimental procedures”), and then further incubated as described in the figure. Protein samples were run on NuPAGE and blotted with anti-FITC to detect transfer of polyP from bis-FAM-P8 to Top1 and with anti-Myc to detect Top1 mobility shift. *C,* shift-up experiment of total protein from gTop1–13Myc–tagged *vtc4*Δ yeast mutant extracted under denaturing conditions (Lysis Buffer (LB) with 2% SDS; see under “Experimental procedures”) or under normal conditions (LB), incubated for 20 min at 30 °C with either 2 mm P65 or P100 or left untreated, run on NuPAGE, and blotted with anti-Myc (to detect Top1), anti-Nsr1, and anti-tubulin (as loading control). *D,* shift-up experiment of total protein from gTop1–13Myc-tagged *vtc4*Δ yeast mutant extracted under native conditions and split in two; one part was denatured by adding SDS to 2% and the other part was kept under native conditions. Protein samples were subsequently treated with increasing concentrations of P100 for 10 min at 30 °C, run on NuPAGE, and blotted with anti-Myc (to detect Top1), anti-Nsr1, and anti-tubulin (α-*Tub*) (as loading control).

One question arises: how could polyP further modify K-PPn proteins? We can imagine two possibilities (Fig. 2*A*): one through the lysine polyphosphorylation of new lysine residues (additional model), or one, chemically less feasible, in which there is a replacement of the shorter polyP chain with the longer one on a particular lysine residue (replacement model). To distinguish between these two models, we performed the following analysis. Non-K-PPn gTop1–GFP was purified from *vtc4*Δ and kept on GFP–TRAP beads. The beads were incubated with a short synthetic polyP of an average chain length of 8 and with fluorescein (FAM) moieties at both ends, herein called bis-FAM-P8 (20). This led to the transfer of FAM to gTop1–GFP as seen by Western blotting (Fig. 2*B*, *lanes 1* and *2*). An anti-FITC antibody was used to detect FAM as they are closely-related molecules. When the same beads were further incubated with P45, we observed a mobility shift of gTop1–GFP but no reduction of the FAM signal (Fig. 2*B*, *lane 3*). This suggests that there is no replacement of bis-FAM-P8 with P45. For further confirmation, we tested whether the mobility shift of K-PPn targets could be further enhanced by denaturation, because this would make additional lysine residues available. We initially tested whether both gTop1–13Myc and Nsr1 can be Lys-polyphosphorylated under denaturing conditions. Proteins were extracted from *vtc4*Δ yeast using no SDS as before or under the denaturing condition of 2% SDS, and then incubated with polyP of average chain length 65 (herein called P65) or P100 (Fig. 2*C*). We observed that both Nsr1 and gTop1–13Myc could still be Lys-polyphosphorylated when denatured. Unlike native proteins, denatured gTop1–13Myc or Nsr1 became readily modified at low P100 concentrations (Fig. 2*D*, *even-numbered lanes*), with this effect more prominent for Top1. These data indicate that the protein denaturation process enhances K-PPn mobility shifts by exposing extra lysine residues, therefore supporting the additional model (Fig. 2*A*). Furthermore, the mobility shift increase depends on the amount of polyP indicating that an early K-PPn event allows further lysine residue(s) to be Lys-polyphosphorylated likely by inducing an opening/unfolding of the polypeptide chain.

### Acidic environments play an essential role in defining polyphosphorylation target regions

**Figure 3.**
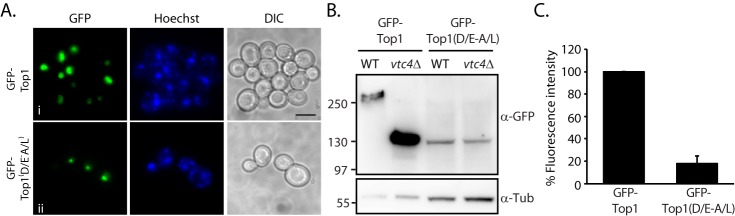
**Glutamic and aspartic acids play an essential role in Top1 polyphosphorylation.** *A*, nuclear distribution of WT yeast exogenously expressing GFP–Top1 and GFP–Top1(D/E-A/L) by confocal microscopy. *DIC*, differential interference contrast. *Scale bar*, 5 μm. *B,* GFP–Top1 and GFP–Top1(D/E-A/L) exogenously expressed in WT yeast were extracted, run on NuPAGE and blotted with anti-GFP and anti-Tubulin (α-*Tub*) (loading control). *C,* GFP–Top1 and GFP–Top1(D/E-A/L) expression levels were measured by FACS. The mean fluorescence intensity of the distribution is given (*n* = 3). All yeast strains are in DDY1810 background. The figures presented are a representation of at least three independent repeats.

As shown, the three-dimensional structure of the target does not seem to play a significant major role in determining K-PPn. Nevertheless, as highlighted in the Introduction, nonenzymatic PTMs do not occur randomly; the physicochemical properties of the amino acids surrounding a specific lysine could still determine the degree of K-PPn. Therefore, we next investigated whether those properties of amino acids enriched in the PASK domain played a direct role in the modification. Previously, we demonstrated that independently mutagenizing either all glutamic or all aspartic acids within the PASK domain did not affect K-PPn (5). However, it is possible that due to their abundance the acidic nature of these amino acids compensates for each other. To test this, we concurrently mutagenized all glutamic and aspartic acids within the PASK domain to alanines and leucines, respectively (Fig. S1; GFP–Top1(D/E-A/L)). We verified that these mutations do not affect Top1 nuclear localization; like GFP–Top1, GFP–Top1(D/E-A/L) correctly localized to the nucleus (Fig. 3*A*). Then we investigated the effect of these mutations on GFP–Top1 K-PPn. We expressed the proteins in both WT and *vtc4*Δ and observed that GFP–Top1(D/E-A/L) shows similar mobility to *vtc4*Δ and WT suggesting a prevention of K-PPn (Fig. 3*B*). Moreover, these mutations also significantly affected the stability of the protein, as the steady-state level of the WT GFP–Top1 is ∼5 times higher than that of the mutant (Fig. 3*C*). Together, these data demonstrate that the acidic environment conferred by the high number of both glutamic and aspartic acids within the PASK domain is essential for K-PPn.

### Vacuolar polyphosphatases Ppn1 and Ppn2 affect polyphosphorylation

**Figure 4.**
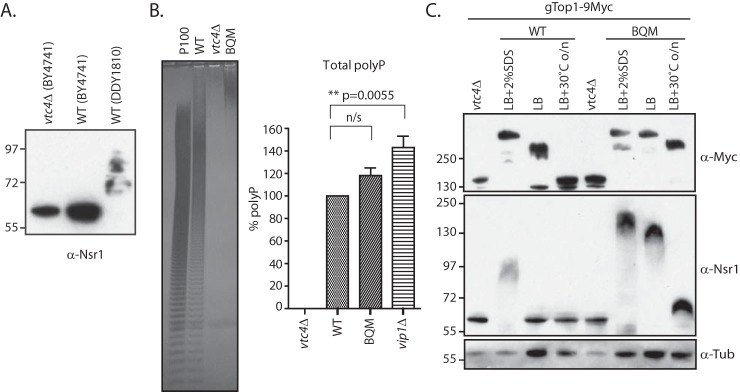
**Effect of phosphatases on polyphosphate and on polyphosphorylation target mobility.** *A,* yeast protein extracts from the strains indicated in the figure were extracted in native buffer (LB+ 2% SDS), run on NuPAGE, and blotted with anti-Nsr1. *B,* total polyP from gTop1–9Myc-tagged *ppn1*Δ*ppn2*Δ*ppx1*Δ*ddp1*Δ BY4741 quadruple mutant (*BQM*). PolyP corresponding to 5 μg of total RNA was fractionated on a 30% polyacrylamide gel, stained by negative staining with DAPI (*left panel*), and quantified by malachite green (*n* = 4, *right panel*). *n/s*, not significant. *C,* gTop1–9Myc endogenously tagged in BY4741 WT (*BWT*) or *ppn1*Δ*ppn2*Δ*ppx1*Δ*ddp1*Δ BY4741 quadruple mutant (*BQM*) backgrounds was extracted under the conditions described in the figure, run on NuPAGE, and blotted with anti-Myc (to detect Top1), anti-Nsr1, and anti-tubulin (α-*Tub*) (as a loading control). All yeast strains are in BY4741 background unless otherwise stated. The figures presented are a representation of at least three independent repeats.

We next investigated the effect of polyP phosphatases on target mobility. Like the exacerbation of mobility shift that occurs during the extraction procedure due to the release of vacuolar polyP, it is possible that the very active polyphosphatases that reside in the vacuole might also affect the K-PPn status of nuclear and cytoplasmic targets. We observed that in the DDY1810 strain, which is depleted of the Pep4 protease required to proteolytically process and activate Ppn1, the mobility of Nsr1 is higher than in the BY4741 strain in which Ppn1 is active (Fig. 4*A*). This suggests that this polyphosphatase can act during cell lysis; as a vacuole-localized endopolyphosphatase (Fig. S2*A*) (14), the presence or absence of Ppn1 should not affect the *in vivo* mobility of either Top1, which has an exclusive nuclear localization, or of Nsr1, which shuttles between the cytoplasm and the nucleus. We therefore decided to investigate the activity of each of the known polyP polyphosphatases. We started by engineering a strain similar to DQM but in the BY4741 background by deleting the four known polyphosphatases (*ppn1*Δ*ppn2*Δ*ppx1*Δ*ddp1*Δ herein called BQM). As expected, and like the DQM, the polyP length in BQM is much higher than that of WT or the *vip1*Δ mutant (Fig. 4*B*, *left panel*). However, the polyP amount, as measured by phosphate content, does not significantly differ from WT (Fig. 4*B*, *right panel*). Next, we compared Top1 and Nsr1 mobilities in WT and BQM under different conditions (Fig. 4*C*) as follows: extraction in lysis buffer (LB); extraction in LB plus 2% SDS, a condition in which the phosphatases are denatured and thus inactive; and extraction in LB with samples incubated overnight at 30 °C to allow polyphosphatases to cleave the phosphoanhydride bonds of polyP. When extracted under denaturing conditions, gTop1–9Myc is equally modified in WT and BQM (Fig. 4*C*, *top panel*). However, gTop1–9Myc quickly loses its mobility shift to nonmodified levels when all the phosphatases are active (*i.e.* WT conditions), which is not observed when all the known polyphosphatases are deleted (*i.e.* in BQM). Upon overnight incubation, gTop1–9Myc in BQM shows a slightly faster migration suggesting that other phosphatases are also able to hydrolyze K-PPn proteins. Analysis of Nsr1 gives slightly different results (Fig. 4*C*, *bottom panel*): in WT, extraction in lysis buffer is generally sufficient for the protein to be depolyphosphorylated; when comparing the mobility of denatured Nsr1 in WT and in BQM, we observe that Nsr1 mobility shift is higher in BQM extracts; and upon overnight incubation, Nsr1 loss of mobility shift is substantial. Taken together, these results suggest that Nsr1 is far more sensitive to polyphosphatases and to other phosphatases than Top1.

**Figure 5.**
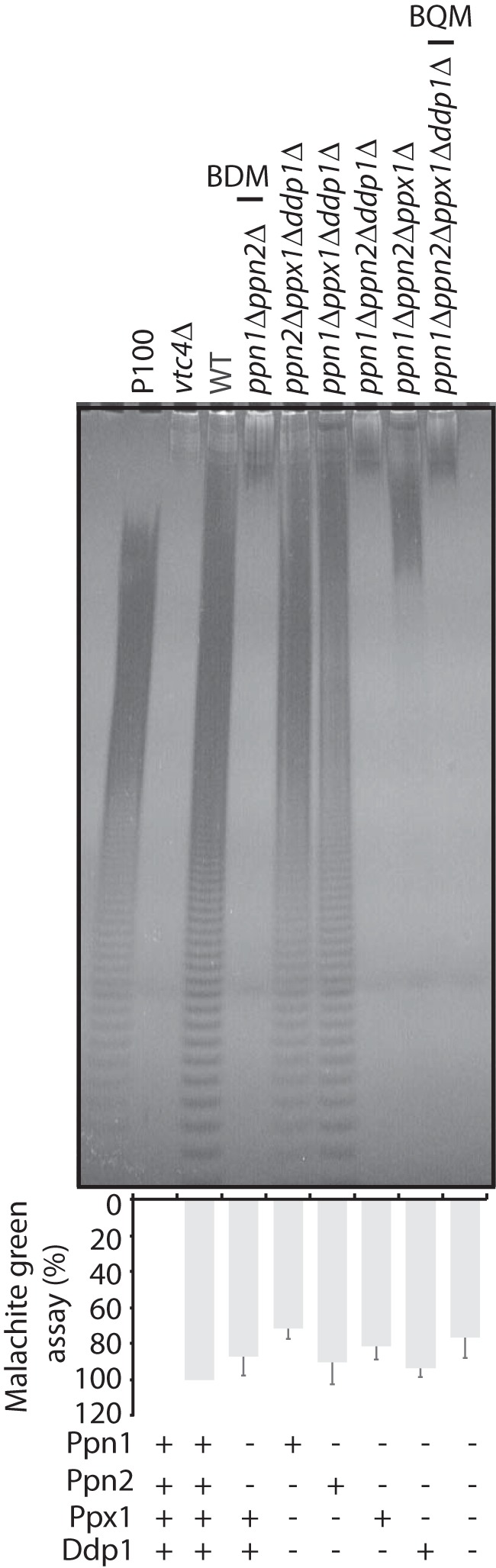
**Polyphosphate from different BY4741 mutants.** Total polyP and respective quantification by malachite green assay from gTop1–9Myc–tagged *ppn1*Δ*ppn2*Δ BY4741 double mutant (*BDM*) and triple and quadruple mutants. PolyP corresponding to 5 μg of total RNA was fractionated on a 30% polyacrylamide gel, stained by negative staining with DAPI (*top panel*), and quantified by malachite green (*n* = 4, *bottom panel*). The figure presented is a representation of four independent repeats.

**Figure 6.**
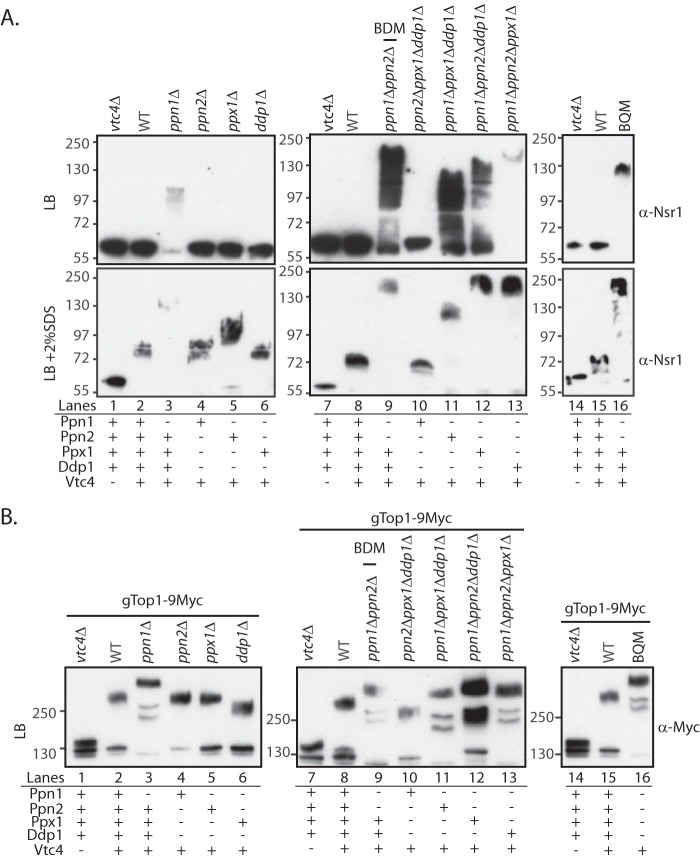
**Top1 and Nsr1 mobility in different BY4741 mutants.** *A,* effect of deleting all known polyphosphatases on Nsr1 mobility. Proteins from different mutants were extracted under native (LB; *top panel*) or denaturing conditions (LB + 2% SDS; *bottom panel*), run on NuPAGE, and blotted with anti-Nsr1. *B,* effect of deleting all known polyphosphatases on Top1 mobility. Proteins from different mutants tagged with gTop1–9Myc were processed as in *A* and blotted with anti-Myc. All yeast strains are in the BY4741 background. The figures presented are a representation of at least three independent repeats.

Next, we analyzed the effect on polyP metabolism and on K-PPn–induced mobility shift of each polyphosphatase individually and in combination. Individually, the polyphosphatase with the greatest impact on polyP chain length was Ppn1; knockout of Ppn1 increased polyP length while no significant impact was observed upon individual deletion of the other polyphosphatases, confirming previously published results (Fig. S2*B*) (21). However, this increase is considerably higher upon simultaneous deletion of Ppn2, the other vacuole-localized endopolyphosphatase (Fig. 5 and Fig. S2, *A* and *B*) (16). Further deleting both Ppx1 and Ddp1 to create the BQM strain did not seem to impact polyP chain length (Fig. 5). These latter enzymes localize mainly to the cytoplasm and the nucleus to a lesser extent (Fig. 2*C*) (22). We next tested the effect of these knockouts on the mobility of both Nsr1 and Top1 (Fig. 6). When extracted under nondenaturing conditions (Fig. 6*A*, *top panel*), Nsr1 showed extreme sensitivity to the presence of Ppn1 because whenever this endopolyphosphatase was present (*all lanes except 3, 9,* and *11–13*), Nsr1 was completely depolyphosphorylated, demonstrating that there is some post-extraction processing by these enzymes. The highest Nsr1 mobility shift observed in the nondenaturing condition is when Ppn1 and Ppn2 are simultaneously deleted (Fig. 6*A*, *lane 9*). Under denaturing conditions where polyphosphatases are inactive (Fig. 6*A*, *bottom panel*), the trend is similar, but there is a clear difference between Nsr1 mobility in WT and *vtc4*Δ that was not seen before (*lanes 1* and *2,* and *7* and *8*). Interestingly, there is also a difference between Nsr1 mobility in BQM under native and denaturing conditions (Fig. 6*A*, *lane 16, top* and *bottom*, respectively) suggesting that other phosphatases are also able to hydrolyze Nsr1 K-PPn. Together, these data demonstrate that several phosphatases exert a post-extraction effect on Nsr1 mobility and degree of K-PPn. Unlike Nsr1, when extracted under nondenaturing conditions, gTop1–9Myc (Fig. 6*B*) showed a considerable mobility difference between WT and *vtc4*Δ (*lanes 1* and *2, 7* and *8,* and *14* and *15*), further demonstrating that K-PPn of Top1 is less sensitive to polyP phosphatases than Nsr1. However, we still observed that Ppn1 does act on Top1 post-extraction, because gTop1–9Myc mobility shift was greatest when Ppn1 is not present (Fig. 6*B*, *lane 3*). The effect of also deleting Ppn2 was negligible, if present (Fig. 6*B*, *lane 9*). However, this effect might be undetectable due to the lack of gel resolution at this high molecular weight. For the same reason, it is difficult to analyze differences between the deletion mutants following gTop1–9Myc extraction under denaturing conditions, as the K-PPn–induced mobility shift is very large for all knockout strains (Fig. S2*D*). In conclusion, these results indicate that polyP phosphatases, in particular Ppn1 and Ppn2, are responsible for hydrolyzing K-PPn on target proteins during the extraction procedures.

### Creating a model yeast strain to study endogenous polyphosphorylation

**Figure 7.**
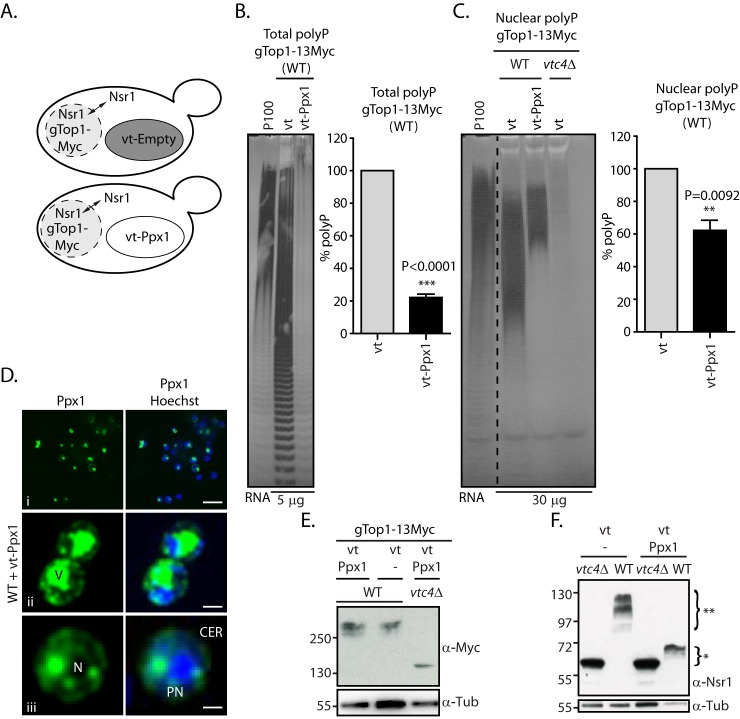
**Engineering a yeast strain to study endogenous polyphosphorylation in DDY1810 WT background.** *A,* schematic representation of WT yeast cells expressing a vacuolar (*vt*) localized Ppx1 (*vt-Ppx1*; *bottom panel*) or an empty vector (*top panel*). The presence of polyP in the vacuole is shown in *dark gray* as seen by EM (31). *B,* total polyP from gTop1–13Myc-tagged WT cells overexpressing empty vector control or vt-Ppx1. PolyP corresponding to 5 μg of total RNA was fractionated on a 30% polyacrylamide gel, stained by negative staining with DAPI (*left*), and quantified by malachite green assay (*right*, *n* = 4). *C,* nuclear polyP of the same strains as in *A*. Nuclear polyP corresponding to 30 μg of total RNA was run as in *B* (*right, n* = 4 (two biological replicates and two technical replicates)). *D*, localization of vt-Ppx1 by immunofluorescence. The vt-Ppx1 is stained in *green* and the nucleus in *blue* (*V,* vacuole; *N,* nucleus; *PN,* perinuclear; *CER,* cortical endoplasmic reticulum; *i scale bar,* 30 μm; *ii scale bar,* 3 μm *iii scale bar,* 1.5 μm. *E,* comparison of Top1 mobility in gTop1–13Myc-tagged *vtc4*Δ and in WT yeast overexpressing empty vector or vt-Ppx1. *F,* comparison of Nsr1 mobility in *vtc4*Δ and in WT yeast overexpressing empty vector or vt-Ppx1. The figures presented are a representation of at least three independent repeats unless otherwise stated. * represents Nsr1 real mobility shift under polyphosphorylation; ** represents Nsr1 mobility shift upon contact with the vacuolar localized polyP.

**Figure 8.**
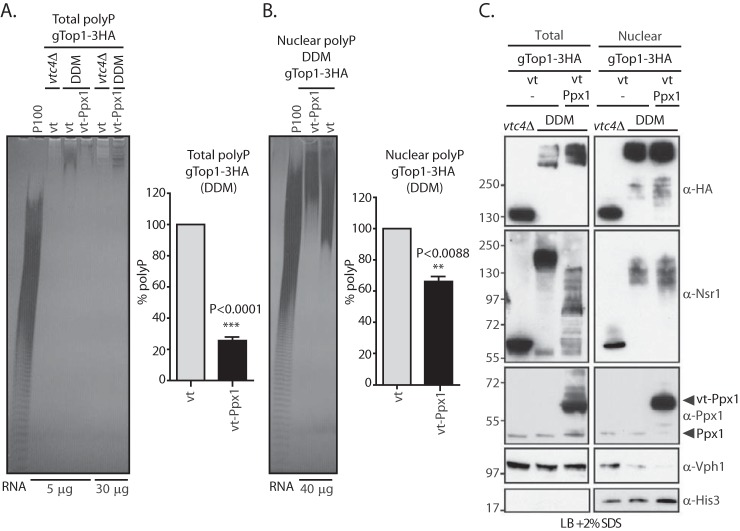
**Engineering a yeast strain to study endogenous polyphosphorylation in DDY1810 *ppn1*Δ*ppn2*Δ background.** *A,* total polyP from gTop1–3HA-tagged *ppn1*Δ*ppn2*Δ cells overexpressing empty vector control or vt-Ppx1 and gTop1–3HA-tagged *vtc4*Δ overexpressing empty vector control. PolyP corresponding to 5 μg of total RNA was fractionated on a 30% polyacrylamide gel, stained by negative staining with DAPI (*left*), and quantified by malachite green assay (*n* = 4). *B,* nuclear polyP of the same strains as in *A*. PolyP was run as in *A* but with polyP corresponding to 40 μg of RNA (*n* = 3 (two biological replicates in which one of the biological replicates has two technical replicates)). *C,* comparison of Top1 and Nsr1 mobility in total protein extracts (*left panel*) and nuclear protein extracts (*right panel*, *n* = 2) from gTop1–3HA-tagged *vtc4*Δ and *ppn1*Δ*ppn2*Δ (*DDM*) overexpressing vt-Ppx1 or empty vector. Protein samples were extracted under denaturing conditions, run on NuPAGE, and blotted with anti-HA (to detect Top1), anti-Nsr1, anti-VPH1 (as a vacuolar marker control), anti-Ppx1, and anti-histone 3 (as a nuclear marker control). All yeast strains are in a DDY1810 background. The figures presented are a representation of at least three independent repeats unless otherwise stated.

Budding yeast is currently the only model available to study K-PPn endogenously, relying on a mobility shift readout on NuPAGE gels. However, as we have demonstrated, both vacuolar polyP and polyphosphatases can influence the modification and mobility shift of K-PPn target proteins during cell lysis. It is therefore imperative to develop a model that eliminates this interference, allowing true readout of endogenous cytosolic/nuclear K-PPn. To eliminate vacuolar polyP, we targeted the exopolyphosphatase Ppx1 to the vacuole by exogenously expressing vt-Ppx1 (Fig. 7*A*) (8). In DDY1810 strains we observed an 80% reduction by PAGE that was quantified by malachite green assay (Fig. 7*B*). Considering that vacuolar polyP accounts for 80% of the total polyP (5), this result suggests that vt-Ppx1 successfully hydrolyzed this confined polyP pool. To verify that the nuclear polyP pool had not been affected by overexpressing vt-Ppx1, nuclear polyP was isolated (Fig. 7*C*). We observed a reduction of the nuclear polyP by about 40% in DDY1810 WT cells overexpressing vt-Ppx1 compared with those expressing a control empty vector. Most of the missing nuclear polyP was of low molecular weight as can be observed by DAPI staining of the polyacrylamide gel (Fig. 7*C*, *left panel*). This reduction in nuclear polyP levels likely reflects post-extraction activity from vt-Ppx1 contamination of the nuclear preparations. Although the overexpressed protein was found correctly mainly in the vacuole, immunofluorescent staining revealed its presence also in the endoplasmic reticulum (Fig. 7*D*), an organelle known to cofractionate with nuclei. Next, we tested the effect of eliminating vacuolar polyP on Top1 and Nsr1 mobility. Top1 is a nuclear protein (24), whereas Nsr1, despite localizing mostly to the nucleus, is known to shuttle between the cytoplasm and the nucleus (25). There was no difference in degree of K-PPn for gTop1–13Myc extracted, under denaturing conditions, from cells expressing empty vector or vt-Ppx1 (Fig. 7*E*). This result suggests that Top1 is endogenously Lys-polyphosphorylated by nuclear polyP and is not sensitive to post-extraction addition of vacuolar polyP. The scenario is different for Nsr1. The mobility shift of Nsr1 is considerably reduced, but still detectable, in WT yeast expressing vt-Ppx1 (Fig. 7*F*, indicated with *). This suggests that the nuclear Nsr1 is endogenously modified by the polyP present in the nucleus. Moreover, Nsr1 is more sensitive to post-extraction contact with vacuolar polyP than Top1 (the shift labeled **). By degrading vacuolar polyP with vt-Ppx1 expression, we had eliminated one source of post-extraction skewing of results. We then considered the highly-active polyphosphatases Ppn1 and Ppn2 (Fig. 6). To gain the truest representation of Nsr1 physiological K-PPn levels, we overexpressed vt-Ppx1 in a DDY1810 *ppn1*Δ*ppn2*Δ strain, herein called DDM. We confirmed that the overexpression strain was devoid of vacuolar polyP by measuring total polyP levels (∼26%) when compared with the same strain expressing the empty vector (Fig. 8*A*). As before, we performed nuclear fractionation and quantified the nuclear polyP. Like WT, we observed that there is a 40% reduction of nuclear polyP in the DDM cells expressing vt-Ppx1 (Fig. 8*B*). In this case, we verified that there was some vacuolar and significant endoplasmic reticulum contamination, noted by the presence of Vph1 (subunit a of vacuolar-ATPase V0 domain) and vt-Ppx1, respectively, in the nuclear fraction, likely explaining the 40% reduction in nuclear polyP levels (Fig. 8*C*). We investigated Top1 and Nsr1 mobility in total and nuclear protein extracts from these cells (Fig. 8*C*). As for the DDY1810 WT strain, gTop1–3HA mobility was not affected in the DDM strain expressing vt-Ppx1. Consistent with our previous analysis, Nsr1 was more sensitive than Top1 to the presence of Ppn1 and Ppn2, as its mobility shift was greater than when Ppn1 and Ppn2 are absent (Fig. 8*C*) than in WT (Fig. 7*F*). This likely represents the physiological extent of Nsr1 K-PPn. By eliminating both vacuolar polyP and vacuolar polyphosphatases, the yeast *ppn1*Δ*ppn2*Δ (DDM) expressing vt-Ppx1 is the ideal strain in which to study K-PPn of target proteins.

## Discussion

### Vacuolar polyP exacerbates polyphosphorylation during protein extraction

K-PPn is a nonenzymatic PTM that results in the covalent attachment of polyP to lysine residues localized in PASK domain-containing proteins. This modification has been mostly characterized in budding yeast (5, 26). Yeast possess a unique polyP physiology; it accumulates inside the acidocalcisome-like (27) vacuole, and it is actively processed by vacuolar polyP phosphatases. This distinctive polyP metabolism coupled with the nonenzymatic nature of K-PPn raises a few questions that our study aimed to address. The polymeric nature of polyP, with a size that ranges from ten to hundreds of phosphate residues in length, suggests that the covalent association of polyP to target proteins would result in an upward smear as seen, for example, with polyubiquitination. However, often this PTM results in a discrete upward mobility shift on NuPAGE, with only one band seen. K-PPn, as with virtually all nonenzymatic PTMs, occurs when the reactive metabolite, in this case polyP, is in direct contact with its targets. Importantly, 80% of yeast polyP resides in the vacuole and is not physiologically available to modify cytoplasmic and nuclear targets. Here, we confirmed that during cell lysis the polyP released from vacuoles can polyphosphorylate not only nonmodified Nsr1 but also increases the mobility shift of previously polyphosphorylated Nsr1. We showed that this occurs by modification of further lysine residues by subsequently added polyP. The initial K-PPn event may lead to an exposure and availability of further targetable lysine residues, perhaps suggesting that K-PPn has a denaturing effect on target proteins. This hypothesis agrees with the observation that K-PPn Top1 is inactive in its capacity to relax supercoiled DNA (5). The ability of vacuolar polyP to successively modify lysines, perhaps until the protein is saturated, could explain why K-PPn in yeast does not lead to a smear on NuPAGE; the polydisperse nature of this modification is especially difficult to define at very high molecular weights.

### PASK domain's physicochemical properties are essential for polyphosphorylation

The PASK domain is characterized by an abundance of the acidic glutamic and aspartic acids, lysines, and serines. Serine residues do not seem to be directly or indirectly required for the modification to occur, because mutagenizing them all to alanine within the PASK domain did not change the capacity of Top1 to be K-PPn (5). When mutagenized individually, glutamic and aspartic acids also appeared not to be important for K-PPn. However, when mutagenized all together with the neutral amino acids alanine and leucine, respectively, Top1 was no longer Lys-polyphosphorylated by polyP, despite maintaining its nuclear localization. Possibly, these acidic amino acids could coordinate polyP, aided by divalent cations such as Mg^2+^, to position the polymer near the target lysine for K-PPn. Upon neutralization of the negative charge of the acidic amino acids through mutagenesis, this role played by the side chain negative charges no longer exists, and the protein cannot be Lys-polyphosphorylated. This resembles other modifications, such as sumoylation, for example, where the acidic residues surrounding the Ubc9 consensus region enhance the efficiency and specificity of the modification (28).

### Vacuolar polyphosphatases Ppn1 and Ppn2 negatively affect polyphosphorylation of nuclear proteins upon extraction

In yeast, there are four known polyP phosphatases, Ppn1 and Ppn2 (14, 16, 29), Ddp1 (30), and Ppx1 (18). Both the exopolyphosphatase Ppx1 and the endopolyphosphatase Ddp1, which are present in the nucleus and the cytoplasm, have previously been shown to hydrolyze the polyP of K-PPn target proteins Nsr1 and Top1 (5). Interestingly, under native conditions, Ppx1 shows a much higher affinity for K-PPn Nsr1 than Top1, both *in vitro* (5) and *in vivo*. One possible explanation is that the conformation of K-PPn Nsr1 exposes polyP more on the surface allowing for easier access of Ppx1 than in the case of Top1. The vacuolar-localized Ppn1 requires protease processing to become active (14), and in a yeast background that has the Pep4 protease deleted, DDY1810, Nsr1, and Top1 show a higher-mobility shift. We therefore investigated the actual effect of the vacuolar polyphosphatases Ppn1 and Ppn2 in the hydrolysis of K-PPn targets. Despite their vacuolar localization and therefore not in direct contact with cytoplasmic and nuclearly-localized K-PPn targets *in vivo*, upon cell lysis, the very active Ppn1 was shown to rapidly hydrolyze target proteins. Deletion of Ppn2 alone did not dramatically change the mobility of protein targets, but its deletion in conjunction with Ppn1 had a synergistic effect on K-PPn. This demonstrates that both can hydrolyze K-PPn targets but that Ppn1 has a much higher affinity for these targets. Our results also demonstrate that besides the polyphosphatases studied here, there are others that also hydrolyze phosphorylated target proteins, as seen by loss of the downward mobility shifts of Nsr1 in the DQM under native conditions (Fig. 4*B*).

### Generation of an ideal strain to study polyphosphorylation in yeast

We have demonstrated that in yeast, upon cell lysis, the abundant vacuolar polyP becomes available to K-PPn target proteins present in the cytoplasm and the nucleus. At the same time, there is also the release from the vacuole of the very active endopolyphosphatases, Ppn1 and Ppn2, having the opposite effect, *i.e.* hydrolysis of K-PPn target proteins. For these reasons, during extraction in WT, the tug–of–war between these two effects results in a mobility shift on NuPAGE of K-PPn target proteins that might not truly reflect the physiological conditions. Despite this drawback, yeast is still the best eukaryotic model in which to study K-PPn. It is a genetically tractable model organism in which the whole biosynthetic pathway for the synthesis and hydrolysis of polyP is known and well-described. Next, yeast contains high levels of polyP, making the only readout so far available to detect K-PPn of targets proteins, the mobility shift on NuPAGE, possible. Higher eukaryotes not only contain much less polyP, but also the enzyme(s) responsible for its synthesis are unknown, making mammalian cell lines an unfriendly model system in which to study K-PPn events for the time being. Taking all this into consideration, an ideal model in which to study this new PTM would be a yeast strain devoid of vacuolar polyP and deleted of Ppn1 and Ppn2. Therefore, we have targeted the exopolyphosphatase Ppx1 to the vacuole in *ppn1*Δ*ppn2*Δ DDY1810 strain and ascertained that it had lost its vacuolar polyP but retained the nuclearly-localized polyP. With only the nuclear polyP present and in the absence of the highly-reactive Ppn1 and Ppn2, we observed that Top1 mobility remains unaltered, implying that it is physiologically fully K-PPn. Interestingly, Nsr1 behavior is different; this target is a lot more sensitive to the presence of vacuolar polyP and of the vacuolar polyphosphatases. Under physiological conditions, Nsr1 might not be fully phosphorylated and upon exposure to extra polyP from the vacuole during the extraction, it might be further modified. Importantly, nuclear preparations from *ppn1*Δ*ppn2*Δ DDY1810-expressing Ppx1 in the vacuole revealed a substantial K-PPn induced Nsr1 mobility shift, highlighting the importance of this newly-created yeast strain to study K-PPn physiological events. The demonstration that nuclear polyP is insensitive to the action of vacuole-localized Ppx1 suggests the existence of two distinct and separate polyP pools. This raises the question of the origin of the polyP nuclear pool. As mentioned in the Introduction, in yeast, polyP is synthesized by the VTC complex mostly by the subcomplex composed of the subunits Vtc1/3/4/5 (32), which is localized in the cytosolic side of the vacuole membrane (23). Upon synthesis, polyP is translocated and accumulates in the vacuole lumen (23). However, the existence of a second VTC subcomplex, Vtc1/2/4/5 (32), which localizes to the endoplasmic reticulum in regions adjacent to the nucleus, suggests a possible direct synthesis of polyP in the nucleus. Another possibility that cannot be excluded is that a portion of newly-synthesized polyP is released in the cytosol and transported into the nucleus complexed with proteins, similarly to ribonucleoprotein transport of mRNA (33). More studies are required to verify these hypotheses and to define how the nuclear pool of polyP is generated.

In the meantime, our freely available strain (*ppn1*Δ*ppn2*Δ DDY1810-expressing vt-Ppx1) will allow us and the community to study protein K-PPn of cytosolic and nuclear targets without the interfering effects, observable upon cell lysis, of the abundant vacuole polyP pool and of the active vacuole Ppn1 and Ppn2 polyphosphatases. Therefore, studies in this strain are closer to the physiological context.

## Experimental procedures

### Yeast strains and culture media

*S. cerevisiae* strains used in this study are isogenic to DDY1810 or BY4741 and are listed in Table S1. Genes were deleted by replacing the entire ORF with the marker cassettes (see Table S2 for PCR primers) (34). The DDY1810 has a deletion on the Pep4 protease that is coincidently required for processing and activating the endopolyphosphatase that cleaves polyP internally, Ppn1 (14). C-terminal genomic insertions for Top1, gTop1–3HA (DDY1810) and gTop1–9Myc (BY4741), were obtained by standard procedures according to Ref. 34 using primers in Table S2. Correct recombination was confirmed by PCR, and correct protein expression was confirmed by Western blotting with anti-HA and Myc antibodies, respectively.

Yeast strains were grown in SC media: 20% glucose, yeast nitrogen base without amino acids (Sigma, Y0626) complemented with complete synthetic media (CM: MPBio-4500-022) if WT; complete synthetic dropout media without uracil (CSM-URA: MPBio-4511-222) if transformed with GPD-vt-Ppx1 vector or pCA384 (GFP–Top1); or complete synthetic dropout media without tryptophan (CSM-TRP: MPBio-4511-022) if transformed with pCA511 (GFP–Top1(D/E-A/L)). Yeast WT cells for lithium acetate transformation were grown on YPD media (Formedia; CCM0210).

Please note that in WT BY4741, overexpression of vt-Ppx1 did not change total polyP levels, whereas in WT DDY1810 it abolished vacuolar polyP, and therefore this strain was used for the experiments.

### Expression constructs

Plasmids for yeast expression were cloned with the oligonucleotides described in Table S2 as follows. 1) pCA82-pYes3-ADH::GFP(TRP) was cloned by digesting pYes3-ADH with SpeI and NotI to remove the Trp gene and insert it into pCA58 (pYes-pADH:GFP(URA); (3)) digested with the same enzymes. 2) pCA384-pYes-ADH::GFP-ScTop1 (GFP–Top1 in Fig. 3) was cloned by digesting pCA61-pYes-ADH::GST-ScTop1 (3) with BamHI and NotI to remove ScTop1 and insert it into pCA58 (3) digested with the same enzymes. 3) pCA511-pYes3-ADH:: GFP-ScTop1 (E-L, D-A within PASK; GFP–Top1(D/E-A/L) in Fig. 3) was cloned with overlap extension PCR; in short, PCR1 was generated by amplifying ScTop1-PASK (E-L, D-A) generated by gene synthesis (IDT) with OC253 and OC254 primers (Table S2), and PCR2 was generated by amplifying pCA61-pYes-ADH::GST-ScTop1 (3) with OC255 and OC92 primers (Table S2). A third PCR was generated using PCR1 and PCR2 as templates first for 10 cycles, without primers and then with OC253 and OC92 (Table S2), and PCR3 was digested with BamHI and NotI and cloned into pCA82 digested with the same enzymes. 4) pGPD-vt-PPX1 (referred vt-Ppx1 in the text) and pGPD-vt (referred to vt or empty vector in the text) were obtained from Ref. 16.

### Antibodies

The antibodies used in this study were as follows: mouse anti-Nsr1 (Abcam, ab4642; 1:5000); mouse anti-Myc (clone 9A10; Santa Cruz Biotechnology, C0613; 1:2000); rat anti-tubulin (Abcam, ab6160; 1:5000); mouse anti-HA (clone HA-7, Sigma, H3663; 1:2000); mouse anti-GFP (Roche Applied Science, 11814460001; 1:2000); rabbit anti-Ppx1 (Abcam, ab225684; 1:2000); rabbit anti-histone 3 (Abcam, ab1791; 1:10,000); mouse anti-VPH1 (clone 10D7A7B2; Abcam, ab113683; 1:5000); goat anti-FITC (Abcam, ab19224; 1:2000); and anti-mouse Alexa Fluor 488 donkey (Thermo Fisher Scientific, A-21202; 1:1000).

### Protein extraction

Yeast cells grown overnight in appropriate minimal media were diluted to OD_600_ = 0.8 and grown for 90 min at 30 °C in 10 ml of fresh media. Cells were pelleted through centrifugation at 4100 × *g* for 3 min, washed once in ice-cold MilliQ water, and frozen at −20 °C. To avoid depolyphosphorylation, it is essential to work quickly, maintain cold cell extracts throughout the entire procedures, use a wide range of phosphatase inhibitors, and keep incubation times to a minimum. Unless otherwise stated, proteins were extracted in ice-cold lysis buffer (LB: 50 mm Tris-HCl, pH 8.0, 150 mm NaCl, 5 mm DTT, phosphatase inhibitor mixture 1; phosphatase inhibitor mixture 1 (PhIC1): 100×: 200 mm imidazole, 100 mm sodium fluoride, 115 mm sodium molybdate, and 100 mm sodium orthovanadate; phosphatase inhibitor mixture 2 (PhIC2): 1000×: 2.5 mm (−)-*p*-bromotetramisole oxalate and 0.5 mm cantharidin; and protease inhibitor mixture (PrIC): from Sigma catalog no. P8215). Cells were vortexed in the presence of glass beads for 30 s to 1 min and homogenates centrifuged at top speed for 5 min at 4 °C. Supernatants were immediately boiled in 4× SDS sample buffer (0.25 m Tris-HCl, pH 6.8, 8% SDS (w/v), 30% glycerol, 0.02% (w/v) bromphenol blue) for 5 min. Proteins were run on 4–12% NuPAGE gels (Invitrogen) and transferred to polyvinylidene difluoride membranes, and Western blotting was performed with the appropriate antibodies. When proteins were extracted under denaturing conditions, two different buffers were used: 2% SDS (LB containing 2% SDS) and 8 m urea buffer (8 m urea, 0.05 m sodium phosphate buffer, pH 7.6, and 0.14 m β-mercaptoethanol). Extraction procedure was as described above.

To verify if during the extraction procedure polyP is able to associate with nonmodified proteins (Fig. 1*A*), 5 ml of gTop1–13Myc (*vtc4*Δ) culture OD_600_ = 0.8 was mixed with a *vip1*Δ (DDY1810), *vtc4*Δ (DDY1810), or WT (DDY1810; DWT) culture of the same OD (1:1 ratio). Mix population cells were spun, washed twice in ice-cold water, frozen at −20 °C, and subsequently extracted in lysis buffer as described above.

For Myc immunoprecipitations, equilibrated beads, Myc-agarose (Sigma A7470), were incubated with normalized protein extracts (500 μl of protein extract (1 μg/μl) with 50 μl of 50% (v/v) beads) with head–over–tail rotation for 1 h at 4 °C. The mixture was centrifuged at 369 × *g* for 1 min and washed four times for 10 min at 4 °C in LB containing 0.75% Triton X-100. Beads were either equilibrated twice in LB (without detergent) and kept at 4 °C until further use (no more than a week) or were resuspended in 2× SDS sample buffer and boiled. Proteins were run on 4–12% NuPAGE gels as above.

To extract total polyP, yeast cells grown overnight were diluted to OD_600_ = 0.8 and grown in 10 ml of SC minimal media for 90 min at 30 °C. Cells were pelleted at 4122 × *g* for 3 min and washed twice in 1 ml of cold MilliQ water. The cell pellet was resuspended in 250 μl of LETS buffer (0.1 m LiCl, 10 mm EDTA, 10 mm Tris-HCl, pH 7.4, and 0.2% SDS) and 250 μl of acidic phenol, pH 4.3 (Sigma; P4682), was added together with the corresponding of 25 μl of glass beads (Sigma; G8772). Cells were turbo-vortexed for 5 min at 4 °C and spun at top speed for 5 min. The top layer was transferred to a fresh tube and extracted with 500 μl of chloroform through turbo-vortexing (5 min at 4 °C) and spun as above. The top layer was transferred to a fresh tube and precipitated with 2.5× ethanol for at least 2 h at −20 °C. Extract was spun as above; supernatant was aspirated, and samples were air-dried before being resuspended in 200 μl of resuspension buffer (RB: 0.1% SDS, 1 mm EDTA, 10 mm Tris-HCl, pH 7.4). Quantification of RNA in polyP extract was determined by reading absorbance at 260 nm in the nanodrop. PolyP was analyzed by PAGE and visualized by negative staining with DAPI according to previously described methods (30).

A similar procedure was used to extract nuclear polyP. Purified nuclei were resuspended in 300 μl of LETS buffer, passed through a 0.2-μm needle four times to shear the DNA, and 300 μl of acidic phenol, pH 4.3, was added. Sample was turbo-vortexed without glass beads following the same procedure as above.

### Shift-up assay

The shift-up experiments were performed in different ways (refer to figure legends for details). The sources of polyP used throughout this assay were as follows: (*a*) purified from yeast polyP (section polyP extraction); (*b*) synthetic polyP of an average size of 45 (P45; Sigma, S4379), 65 (P65; Sigma, S6253), and 100 (P100; kindly provided by Dr. Toshikazu Shiba, RegeneTiss Co., Japan), and bis-FAM-P8 (20); (*c*) nonpurified, in this case yeast cultures from *vtc4*Δ and ones containing polyP were mixed in a 1:1 ratio (OD_600_ = 0.8), spun at 4100 × *g*, washed once in ice-cold water, and extracted as described above under “Protein extraction.” The substrates for this assay were as follows: 1) protein extracts from the different panel of yeast mutants; and 2) unmodified purified proteins, purified from *vtc4*Δ on Myc-agarose beads or GFP-trap (Chromotek-gta-10; refer to figure legends). When the assay was performed with total protein extracts, 20 μl of protein extract (1 μg/μl) was incubated with 5 μl of 10 mm P45, P65, or P100 (2 mm) for 20 min at 30 °C, and 4× SDS sample buffer was added, samples were boiled, resolved by NuPAGE, and immunoblotted with appropriate antibodies. When the assay was performed on Myc-agarose or GFP-trap–purified proteins beads, polyP was incubated with 15 μl of bead bed volume in a total volume of 50 μl of RB buffer for 20 min at 30 °C with head–over–tail rotation, in the following concentrations: 5 μl of 10 mm P45, P100 (2 mm), 0.5 mm bis-FAM-P8 or 2 μl of total yeast polyP (1.5 μg/μl of RNA). After incubation, beads were washed three times in LB buffer, boiled in 2× SDS sample buffer, resolved by NuPAGE, and immunoblotted with appropriate antibodies.

### PolyP quantification by malachite green assay

We used the ability of malachite green, molybdate, and free orthophosphate to form a complex that absorbs light of a wavelength between 620 and 640 nm to determine polyP amounts in yeast. PolyP corresponding to 0.8 μg of RNA was converted to orthophosphate by incubation by boiling in 250 μl of 0.3 m perchloric acid for 30 min. Samples were cooled on ice for 2 min, and 250 μl of malachite green reagent (15 mg of malachite green, 100 mg of sodium molybdate, 25 μl of Triton X-100, 2.82 ml of 38% hydrochloric acid in 50 ml water) was added. Samples were incubated 30 min at 30 °C; 150 μl was dispensed in triplicate on a 96-well plate, and absorbance was measured on a Tecan plate-reader fluorimeter at 640 nm. The concentration of hydrolyzed orthophosphates was compared with a P_i_ standard calibration curve. Each sample was measured in triplicate, and the baseline blank was always the reading from *vtc4*Δ. Each experiment is an average of at least three biological replicates.

### S. cerevisiae nuclei fractionation

Nuclei fractionation was adapted from the Hahn laboratory at Fred Hutchinson Cancer Research Center. In summary, yeast cells were grown in 2 liters of YPD (Formedium), supplemented with 3% (w/v) glucose at 30 °C until OD_600_ = 3–5 (∼30 g of cells). When yeast cells had been transformed with pGDP–vt-Ppx1, cells were grown the same way but in minimal media containing 3% (w/v) glucose and without uracil. Cells were pelleted at 4100 × *g* for 10 min, resuspended in 35 ml of 50 mm Tris-HCl, pH 7.5, and 30 mm DTT, and incubated 15 min at 30 °C. Cells were pelleted at 4100 × *g* for 10 min and resuspended in 20 ml of YPD containing 1 m sorbitol (YPDS), and an additional 15 ml of 2 m sorbitol was added. Cells were transformed into spheroblasts with 200 μl of zymolyase (USBiological, Zymolyase 100T-Z1004) and incubated at 30 °C with gentle occasional swirling. When 80% of the cells were transformed into spheroblasts, 100 ml of YPDS was added, and cells were pelleted at 4100 × *g* for 10 min. Cells were resuspended in 250 ml of YPDS and allowed to recover at 30 °C for 30 min. Cells were pelleted at 4100 × *g* for 10 min and washed twice in 200 ml of ice-cold YPDS. Cells were pelleted at 4100 × *g* at 4 °C for 10 min and resuspended in 250 ml of ice-cold 1 m sorbitol. Cells were pelleted as above and resuspended in 100 ml of Buffer N (25 mm K_2_SO_4_, 30 mm Hepes, pH 7.6, 5 mm MgSO_4_, 1 mm EDTA, 10% glycerol, 0.5% Nonidet P-40, 7.2 mm spermidine, 3 mm DTT, and 1:500 PrIC). Cells were subjected to 4–6 strokes in a type A “tight” Dounce on ice, transferred to 50-ml centrifuge tubes, and pelleted at 230 × *g* for 10 min at 4 °C. Supernatant was recovered and spun again at 390 × *g* for 10 min at 4 °C. Supernatant was transferred to a clean centrifuge tube and spun at 1910 × *g* for 10 min at 4 °C. Supernatant was further pelleted at 4303 × *g* for 10 min at 4 °C to recover the nuclei. Nuclei were frozen at −80 °C.

### FACS

Cells were grown in SC minimal medium to logarithmic phase. Just before measurement, 50 μl of the cell suspension was added to 950 μl of TBS, vortexed, and immediately analyzed using an LSRII flow cytometer (BD Biosciences). 20,000 events were recorded for each sample, and the mean fluorescence value was used to estimate protein expression levels.

### Immunofluorescence and fluorescence microscopy

To determine vt-Ppx1 localization, in *ppn1*Δx*ppn2*Δ gTop1-3HA-tagged cells expressing GPD-vt-Ppx1 we performed immunofluorescence with anti-Ppx1 antibody. Cells were grown overnight in Sc media without uracil at 30 °C, diluted in fresh media to OD_600_ 0.6, and grown for 90 min. Cells were spun at 4100 × *g* for 3 min, resuspended in 2 ml of softening buffer (50 mm Tris-HCl, pH 7.5, and 30 mm DTT), and incubated for 15 min at 30 °C. Cells were pelleted at 4100 × *g* for 3 min, resuspended in 2 ml of a solution containing 2:1.5 of YPDS and 2 m sorbitol and 20 μl of zymolyase (USBiological, Zymolyase 100T-Z1004), and incubated at 30 °C with occasional gentle swirling. When 80% of the cells were transformed into spheroblasts, formaldehyde was added to 4%, and cells were further incubated for 60 min at 30 °C. Cells were spun at 390 × *g* for 2 min, carefully resuspended in 0.5 ml of Buffer A (100 mm Tris-HCl, pH 8, 1 m sorbitol) with 0.1% (v/v) SDS, and incubated at room temperature for 10 min. Cells were spun as above, gently washed twice in Buffer A, resuspended in 0.5 ml of Buffer B (50 mm Tris-HCl, pH 8, 150 mm NaCl, 0.1% (v/v) Tween 20, 1% (w/v) nonfat dried milk, 0.5 mg/ml BSA), and incubated at room temperature 15 min to block. After this period, 1 μl of anti-Ppx1 (1:500) antibody was added, and cells were incubated for 90 min at room temperature with head–to–tail rotation. Cells were spun at 390 × *g* for 2 min and washed five times in Buffer B. After the last wash, 0.5 μl of mouse Alexa 488 antibody was added to 500 μl of cell suspension and incubated for 60 min in the dark at room temperature with head–over–tail rotation. Cells were washed as above with two extra washes with PBS, incubated in PBS (2.5 μg/ml Hoechst) for 20 min in the dark, and washed twice again in PBS. Cells were mounted on microscope slides and observed in a spinning disc confocal microscope-Ultra VoxII with a ×100 CFI Plan Apochromat VC 1.4 lens and a Hamamatsu C9100–13 EMCCD camera. Pictures were collected using Velocity software (PerkinElmer Life Sciences) and treated with ImageJ software.

For observation of the polyP phosphatase localizations, cells were grown overnight in Sc minimal media at 30 °C, diluted to OD_600_ 0.6 in fresh media, grown for 60 min, after which DAPI (2.5 μg/ml) was added, and cells were grown for an extra 30 min before being harvested by centrifugation. Cells were resuspended in 50 μl of the same media and mounted onto a microscopy slide. Cells were observed on a widefield microscope, Zeiss Axio Imager with an EC Plan Neofluar ×100 1.3 NA lens, and an QImaging Retiga EXi grayscale CCD camera. Pictures were collected using OpenPlan software and treated with ImageJ software.

### Statistics

Tests for significance of observed differences were performed by an unpaired Student's *t* test, and *error bars* represent the standard deviation. *p* values differences higher than 0.1 were considered as not significant.

## Author contributions

C. A. and Y. D. conceptualization; C. A., Y. J., H. P., and S. T. data curation; C. A. and A. S. formal analysis; C. A. supervision; C. A. validation; C. A. investigation; C. A. methodology; C. A. and A. S. writing-original draft; C. A. and A. S. project administration; C. A., Y. D., Y. J., H. P., S. T., J. S., H. J. J., and A. S. writing-review and editing; A. S. funding acquisition; J. S., N. S., and H. J. J. providing material.

## Footnotes

4 The abbreviations used are:

PTM: post-translational modification
K-PPn: lysine polyphosphorylation
polyP: inorganic polyphosphate
DAPI: 4,6-diamidino-2-phenylindole
FAM: fluorescein
LB: lysis buffer
vt: empty vector.

## References

[1] Khoury G. A., Baliban R. C., and Floudas C. A. (2011) Proteome-wide post-translational modification statistics: frequency analysis and curation of the Swiss-Prot database. Sci. Rep. 1, srep00090 10.1038/srep00090 22034591 PMC3201773

[2] Cloos P. A., and Christgau S. (2002) Non-enzymatic covalent modifications of proteins: mechanisms, physiological consequences and clinical applications. Matrix Biol. 21, 39–52 10.1016/S0945-053X(01)00188-3 11827791

[3] Gould N., Doulias P. T., Tenopoulou M., Raju K., and Ischiropoulos H. (2013) Regulation of protein function and signaling by reversible cysteine *S*-nitrosylation. J. Biol. Chem. 288, 26473–26479 10.1074/jbc.R113.460261 23861393 PMC3772194

[4] Martínez-Ruiz A., Araújo I. M., Izquierdo-Álvarez A., Hernansanz-Agustín P., Lamas S., and Serrador J. M. (2013) Specificity in *S*-nitrosylation: a short-range mechanism for NO signaling? Antioxid. Redox Signal. 19, 1220–1235 10.1089/ars.2012.5066 23157283 PMC3785806

[5] Azevedo C., Livermore T., and Saiardi A. (2015) Protein polyphosphorylation of lysine residues by inorganic polyphosphate. Mol. Cell 58, 71–82 10.1016/j.molcel.2015.02.010 25773596

[6] Azevedo C., and Saiardi A. (2016) Why always lysine? The ongoing tale of one of the most modified amino acids. Adv. Biol. Regul. 60, 144–150 10.1016/j.jbior.2015.09.008 26482291

[7] Gray M. J., Wholey W. Y., Wagner N. O., Cremers C. M., Mueller-Schickert A., Hock N. T., Krieger A. G., Smith E. M., Bender R. A., Bardwell J. C., and Jakob U. (2014) Polyphosphate is a primordial chaperone. Mol. Cell 53, 689–699 10.1016/j.molcel.2014.01.012 24560923 PMC3996911

[8] Smith S. A., Mutch N. J., Baskar D., Rohloff P., Docampo R., and Morrissey J. H. (2006) Polyphosphate modulates blood coagulation and fibrinolysis. Proc. Natl. Acad. Sci. U.S.A. 103, 903–908 10.1073/pnas.0507195103 16410357 PMC1347979

[9] Morrissey J. H., Choi S. H., and Smith S. A. (2012) Polyphosphate: an ancient molecule that links platelets, coagulation, and inflammation. Blood 119, 5972–5979 10.1182/blood-2012-03-306605 22517894 PMC3383012

[10] Brown M. R., and Kornberg A. (2004) Inorganic polyphosphate in the origin and survival of species. Proc. Natl. Acad. Sci. U.S.A. 101, 16085–16087 10.1073/pnas.0406909101 15520374 PMC528972

[11] Rao N. N., Gómez-García M. R., and Kornberg A. (2009) Inorganic polyphosphate: essential for growth and survival. Annu. Rev. Biochem. 78, 605–647 10.1146/annurev.biochem.77.083007.093039 19344251

[12] Zhang H., Gómez-García M. R., Brown M. R., and Kornberg A. (2005) Inorganic polyphosphate in *Dictyostelium discoideum*: influence on development, sporulation, and predation. Proc. Natl. Acad. Sci. U.S.A. 102, 2731–2735 10.1073/pnas.0500023102 15701689 PMC549442

[13] Lander N., Ulrich P. N., and Docampo R. (2013) *Trypanosoma brucei* vacuolar transporter chaperone 4 (TbVtc4) is an acidocalcisome polyphosphate kinase required for *in vivo* infection. J. Biol. Chem. 288, 34205–34216 10.1074/jbc.M113.518993 24114837 PMC3837161

[14] Sethuraman A., Rao N. N., and Kornberg A. (2001) The endopolyphosphatase gene: essential in *Saccharomyces cerevisiae*. Proc. Natl. Acad. Sci. U.S.A. 98, 8542–8547 10.1073/pnas.151269398 11447286 PMC37472

[15] Shi X., and Kornberg A. (2005) Endopolyphosphatase in *Saccharomyces cerevisiae* undergoes post-translational activations to produce short-chain polyphosphates. FEBS Lett. 579, 2014–2018 10.1016/j.febslet.2005.02.032 15792812

[16] Gerasimaitė R., and Mayer A. (2017) Ppn2, a novel Zn^2+^-dependent polyphosphatase in the acidocalcisome-like yeast vacuole. J. Cell Sci. 130, 1625–1636 10.1242/jcs.201061 28302909

[17] Losito O., Szijgyarto Z., Resnick A. C., and Saiardi A. (2009) Inositol pyrophosphates and their unique metabolic complexity: analysis by gel electrophoresis. PLoS ONE 4, e5580 10.1371/journal.pone.0005580 19440344 PMC2680042

[18] Wurst H., Shiba T., and Kornberg A. (1995) The gene for a major exopolyphosphatase of *Saccharomyces cerevisiae*. J. Bacteriol. 177, 898–906 10.1128/jb.177.4.898-906.1995 7860598 PMC176681

[19] Onnebo S. M., and Saiardi A. (2009) Inositol pyrophosphates modulate hydrogen peroxide signalling. Biochem. J. 423, 109–118 10.1042/BJ20090241 19614566

[20] Singh J., Steck N., De D., Hofer A., Ripp A., Captain I., Keller M., Wender P. A., Bhandari R., and Jessen H. J. (2019) A phosphoramidite analogue of cyclotriphosphate enables iterative polyphosphorylations. Angew. Chem. Int. Ed. Engl. 58, 3928–3933 10.1002/anie.201814366 30681761

[21] Ogawa N., DeRisi J., and Brown P. O. (2000) New components of a system for phosphate accumulation and polyphosphate metabolism in *Saccharomyces cerevisiae* revealed by genomic expression analysis. Mol. Biol. Cell 11, 4309–4321 10.1091/mbc.11.12.4309 11102525 PMC15074

[22] Huh W.-K., Falvo J. V., Gerke L. C., Carroll A. S., Howson R. W., Weissman J. S., and O'Shea E. K. (2003) Global analysis of protein localization in budding yeast. Nature 425, 686–691 10.1038/nature02026 14562095

[23] Gerasimaitė R., Sharma S., Desfougères Y., Schmidt A., and Mayer A. (2014) Coupled synthesis and translocation restrains polyphosphate to acidocalcisome-like vacuoles and prevents its toxicity. J. Cell Sci. 127, 5093–5104 10.1242/jcs.159772 25315834

[24] Muller M. T., Pfund W. P., Mehta V. B., and Trask D. K. (1985) Eukaryotic type I topoisomerase is enriched in the nucleolus and catalytically active on ribosomal DNA. EMBO J. 4, 1237–1243 10.1002/j.1460-2075.1985.tb03766.x 2988941 PMC554330

[25] Lee W. C., Xue Z. X., and Mélèse T. (1991) The NSR1 gene encodes a protein that specifically binds nuclear localization sequences and has two RNA recognition motifs. J. Cell Biol. 113, 1–12 10.1083/jcb.113.1.1 1706724 PMC2288927

[26] Bentley-DeSousa A., Holinier C., Moteshareie H., Tseng Y.-C., Kajjo S., Nwosu C., Amodeo G. F., Bondy-Chorney E., Sai Y., Rudner A., Golshani A., Davey N. E., and Downey M. (2018) A screen for candidate targets of lysine polyphosphorylation uncovers a conserved network implicated in ribosome biogenesis. Cell Rep. 22, 3427–3439 10.1016/j.celrep.2018.02.104 29590613

[27] Moreno S. N., and Docampo R. (2013) Polyphosphate and its diverse functions in host cells and pathogens. PLoS Pathog. 9, e1003230 10.1371/journal.ppat.1003230 23658515 PMC3642070

[28] Yang S.-H., Galanis A., Witty J., and Sharrocks A. D. (2006) An extended consensus motif enhances the specificity of substrate modification by SUMO. EMBO J. 25, 5083–5093 10.1038/sj.emboj.7601383 17036045 PMC1630412

[29] Kumble K. D., and Kornberg A. (1996) Endopolyphosphatases for long chain inorganic polyphosphate in yeast and mammals. J. Biol. Chem. 271, 27146–27151 10.1074/jbc.271.43.27146 8900207

[30] Lonetti A., Szijgyarto Z., Bosch D., Loss O., Azevedo C., and Saiardi A. (2011) Identification of an evolutionarily conserved family of inorganic polyphosphate endopolyphosphatases. J. Biol. Chem. 286, 31966–31974 10.1074/jbc.M111.266320 21775424 PMC3173201

[31] Azevedo C., and Saiardi A. (2014) Functions of inorganic polyphosphates in eukaryotic cells: a coat of many colours. Biochem. Soc. Trans. 42, 98–102 10.1042/BST20130111 24450634

[32] Hothorn M., Neumann H., Lenherr E. D., Wehner M., Rybin V., Hassa P. O., Uttenweiler A., Reinhardt M., Schmidt A., Seiler J., Ladurner A. G., Herrmann C., Scheffzek K., and Mayer A. (2009) Catalytic core of a membrane-associated eukaryotic polyphosphate polymerase. Science 324, 513–516 10.1126/science.1168120 19390046

[33] Song M. S., Moon H. C., Jeon J. H., and Park H. Y. (2018) Neuronal messenger ribonucleoprotein transport follows an aging Lévy walk. Nat. Commun. 9, 344 10.1038/s41467-017-02700-z 29367597 PMC5783941

[34] Janke C., Magiera M. M., Rathfelder N., Taxis C., Reber S., Maekawa H., Moreno-Borchart A., Doenges G., Schwob E., Schiebel E., and Knop M. (2004) A versatile toolbox for PCR-based tagging of yeast genes: new fluorescent proteins, more markers and promoter substitution cassettes. Yeast. 21, 947–962 10.1002/yea.1142 15334558

